# Theoretical Analysis of the Local Field Potential in Deep Brain Stimulation Applications

**DOI:** 10.1371/journal.pone.0059839

**Published:** 2013-03-28

**Authors:** Scott F. Lempka, Cameron C. McIntyre

**Affiliations:** Department of Biomedical Engineering, Cleveland Clinic Foundation, Cleveland, Ohio, United States of America; University Medical Center Groningen UMCG, The Netherlands

## Abstract

Deep brain stimulation (DBS) is a common therapy for treating movement disorders, such as Parkinson’s disease (PD), and provides a unique opportunity to study the neural activity of various subcortical structures in human patients. Local field potential (LFP) recordings are often performed with either intraoperative microelectrodes or DBS leads and reflect oscillatory activity within nuclei of the basal ganglia. These LFP recordings have numerous clinical implications and might someday be used to optimize DBS outcomes in closed-loop systems. However, the origin of the recorded LFP is poorly understood. Therefore, the goal of this study was to theoretically analyze LFP recordings within the context of clinical DBS applications. This goal was achieved with a detailed recording model of beta oscillations (∼20 Hz) in the subthalamic nucleus. The recording model consisted of finite element models of intraoperative microelectrodes and DBS macroelectrodes implanted in the brain along with multi-compartment cable models of STN projection neurons. Model analysis permitted systematic investigation into a number of variables that can affect the composition of the recorded LFP (e.g. electrode size, electrode impedance, recording configuration, and filtering effects of the brain, electrode-electrolyte interface, and recording electronics). The results of the study suggest that the spatial reach of the LFP can extend several millimeters. Model analysis also showed that variables such as electrode geometry and recording configuration can have a significant effect on LFP amplitude and spatial reach, while the effects of other variables, such as electrode impedance, are often negligible. The results of this study provide insight into the origin of the LFP and identify variables that need to be considered when analyzing LFP recordings in clinical DBS applications.

## Introduction

While a debate continues on the exact mechanisms producing the motor symptoms of Parkinson’s disease (PD), one current hypothesis is that symptoms arise at least partially from hypersynchronous neural activity in several nuclei of the BG, including the subthalamic nucleus (STN) and the internal segment of the globus pallidus (GPi) [1]. Electrophysiological local field potential (LFP) recordings with intraoperative microelectrodes or deep brain stimulation (DBS) macroelectrodes, have shown prominent oscillatory activity within a specific frequency range, e.g. 13–30 Hz, termed the beta frequency band. This beta-band activity is temporally coupled between the STN and GPi, as well as between these nuclei and various cortical regions [2]. The hypothesis that PD motor symptoms, such as bradykinesia and rigidity, are attributed to beta-band hypersynchrony in the BG is supported by the disruption of these oscillations from voluntary movement and dopamine replacement therapies [1]–[5]. It is also believed that DBS may relieve motor symptoms by disrupting this beta hypersynchrony [6]–[8], although this trend has not been observed in all studies [9], [10].

The LFP is complementary to action potential information and single-unit and multi-unit recordings have demonstrated exaggerated activity and synchrony in the STN that are often coupled to oscillations in the LFP [5], [11], [12]. These observations are consistent with the concept that the LFP reflects synchronized activity in a population of local neurons and their inputs [13]. Beta oscillations can exist throughout the entire STN, but a higher degree of beta synchrony is often observed near the dorsolateral border of the STN [5], [11], [12], [14] and allows for localization of the STN via intraoperative LFP recordings [15], [16]. Clinical outcomes of STN DBS have been positively correlated with the spatial extent and degree of beta hypersynchrony within the STN [13], [14]. The difference in depth between the initial increase in beta activity near the dorsolateral border of the STN and the center of the active DBS contact has been positively correlated with the therapeutic stimulation amplitude and negatively correlated with the overall patient outcome [17]. Therefore, it may be possible to optimize DBS electrode placement and stimulation parameter settings using LFP recordings [17], [18]. Furthermore, because beta-band hypersynchrony in the BG exists chronically, LFP recordings from chronically-implanted DBS electrodes have been proposed as a possible control signal for closed-loop control of DBS [19]–[22].

Although LFP signal analysis is widely utilized in clinical applications, the origin of the recorded LFP is poorly understood. Perhaps the single largest unanswered question is the spatial scale or reach of the LFP. Based on experimental and theoretical studies, it is widely accepted that single-unit recordings only detect neurons within approximately 100 µm of the recording microelectrode [23], [24]. However, defining the spatial dimensions of the LFP in the brain is a matter of debate. It is not clear if LFP recordings represent the activity of small and local neuron populations or large and distributed populations. Several studies suggest the LFP extends only a few hundred microns [25]–[27], while contradictory experimental evidence suggests the LFP can extend several millimeters [28]–[30].

The clinical applications described above exploit the spatial dimensions of the LFP and relative changes in its frequency content. Therefore, successful interpretation of LFP recordings requires a sufficient understanding of the source, recording volume, and potential experimental variables that may affect the composition of the LFP (e.g. electrode geometry, recording configuration, electrode-tissue interface impedance). These questions have largely remained unanswered because they are difficult to address experimentally, and so several groups have attempted to shed light on some of these issues with both analytical and computational techniques [31]–[35]. Linden et al., [33] showed that the spatial reach of the LFP is not simple or stationary but depends on a number of factors, such as neuron morphology, synaptic distribution, and correlation in synaptic activity. Therefore, the spatial reach of the LFP will, in general, not be static within a given experiment. For example, when recording beta-band activity in the STN, drug treatment or electrical stimulation produce changes in neural activity (measured as differences in power) that will alter the spatial reach of the LFP.

Although, previous theoretical studies have improved our understanding of LFP recordings, these theoretical analyses were limited by a number of assumptions (e.g. simple electrostatic and homogeneous conducting media, no representation of the electrode-electrolyte interface or recording electronics). For any general model of LFP activity to successfully describe the source and spatial extent of the LFP, it must account for both physiological (strength, spatial extent, and symmetry of activation in the neural substrate) and technical factors (e.g. electrode characteristics and reference site) [30]. Therefore, the goal of this study was to develop and evaluate a detailed recording model of beta-band activity in the STN. The recording model consisted of frequency-dependent finite element models (FEM) of intraoperative microelectrodes and clinical DBS leads implanted in the brain along with multi-compartment models of STN projection neurons placed near the recording electrode(s). The model infrastructure permitted systematic characterization of numerous variables and their effects on clinical LFP recordings: electrode size; electrode impedance; recording configuration; and filtering effects of the brain, electrode-electrolyte interface (EEI), and recording electronics. The results of this study suggest that the spatial reach of the LFP can extend several millimeters. Electrode geometry and recording configuration also had a substantial effect on LFP amplitude and spatial reach, while the effects of other variables, such as electrode impedance, were often negligible. Preliminary results of this study have been previously reported [36].

## Methods

**Figure 1:**
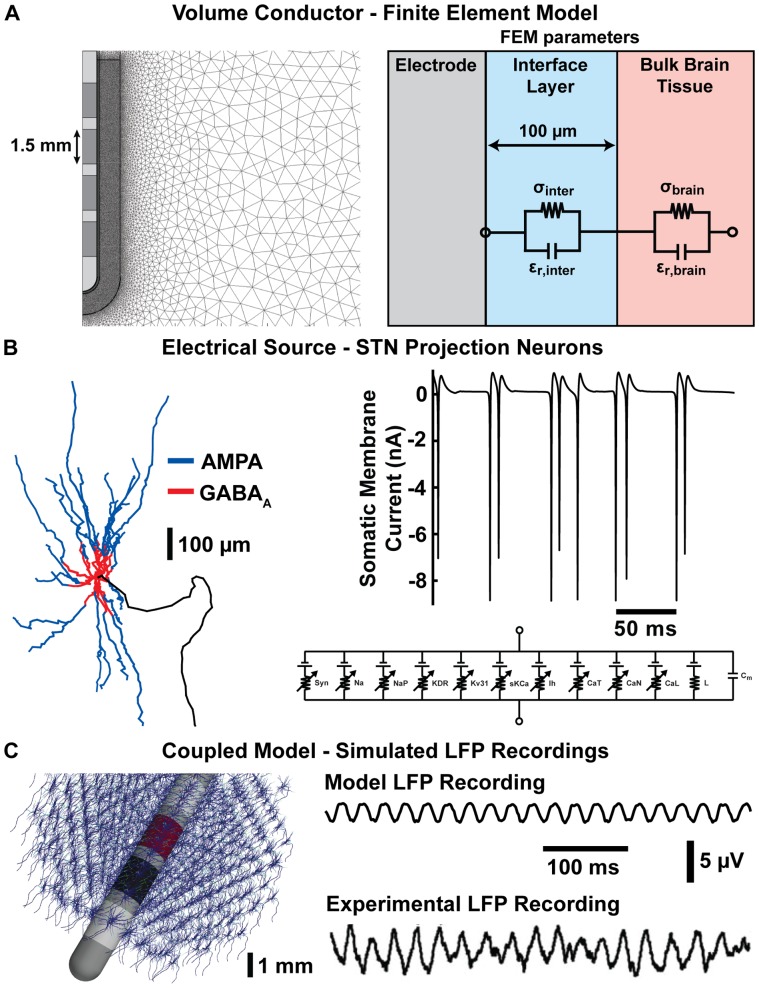
Infrastructure of the LFP recording model. (A) Volume conductor model of a DBS electrode implanted in the brain. The figure on the left shows an example of the finite element mesh with an increased nodal density near the electrode surface. The figure on the right is a schematic showing the standard parameters of the FEM. σ_inter_ and ε_r,inter_ represent the conductivity and relative permittivity of the interface layer whose values were determined from optimizing the FEM parameters relative to in vivo EIS data (see Methods). σ_brain_ and ε_r,brain_ represent the conductivity and relative permeability of the bulk brain tissue, respectively. (B) Electrical source model of STN projection neurons. The figure on the left shows the geometry of the neuron model along with the spatial distribution of the excitatory (AMPA) and inhibitory (GABA_A_) synaptic inputs. The plot on the right shows an example of the typical somatic transmembrane currents generated from the synaptic inputs. The circuit diagram on the bottom right shows an example of the ion channel mechanisms incorporated in the neuron model. (C) The figure on the left shows a DBS electrode surrounded by several hundred STN projection neurons. The voltage trace on the top right shows an example bipolar DBS LFP recording generated with the coupled neuron-FEM model. The voltage trace on the bottom right is an experimental DBS LFP recording adapted from [2].

We developed a detailed computational model of subcortical LFP recordings consisting of two main components: 1) volume conductor models of the recording electrodes (microelectrode or DBS macroelectrode) implanted in the brain, and 2) biophysical models of the individual STN projection neurons. These components were coupled with a reciprocity-based solution to simulate the LFP (Fig. 1). The model infrastructure developed in this study allowed for investigation into a large number of variables that may determine the composition of the recorded LFP.

### Modeling Infrastructure

#### Volume conductor model

The first component of the recording model was a FEM of the recording electrodes implanted in the brain. Two different FEM geometries were developed to represent LFP recordings with either microelectrodes or macroelectrodes. Two-dimensional axisymmetric FEMs were constructed in COMSOL Multiphysics (COMSOL, Burlington, MA) that consisted of a 5×5 cm box. Each model contained a 100 µm-thick interface layer immediately adjacent to the recording electrode(s) to represent electrical inhomogeneities (e.g. edema or tissue encapsulation). To ensure model accuracy, increased mesh densities were used near each electrode (Fig. 1 A).

To investigate LFP recordings with macroelectrodes, we developed a volume conductor model of a DBS electrode implanted in the STN. The electrode had the dimensions of the Medtronic 3389 (Medtronic, Inc., Minneapolis, MN) DBS lead. The DBS lead had four contacts that were 1.27 mm in diameter and 1.5 mm tall (corresponding surface area of 5.98 mm^2^) with 0.5 mm spacing between each electrode. According to common practice, these four electrodes were numbered 0–3, with contact 0 (C0) and contact 3 (C3) as the most distal and proximal electrodes, respectively.

To investigate LFP recordings with microelectrodes, we developed a volume conductor model of a typical intraoperative recording microelectrode (model 5005 Z, FHC Inc., Bowdoinham, ME). The electrode geometry consisted of a microelectrode extending 10 mm from the distal end of a cannula. The microelectrode was 125 µm in diameter and the cannula had an outer diameter of 0.56 mm. The microelectrode tip had an approximate tip angle of 16 degrees with a 50 µm exposure (surface area of 1250 µm^2^) and the distal end of the cannula had a 1 mm tall exposure representing the reference electrode (surface area of 1.26 mm^2^).

**Figure 2:**
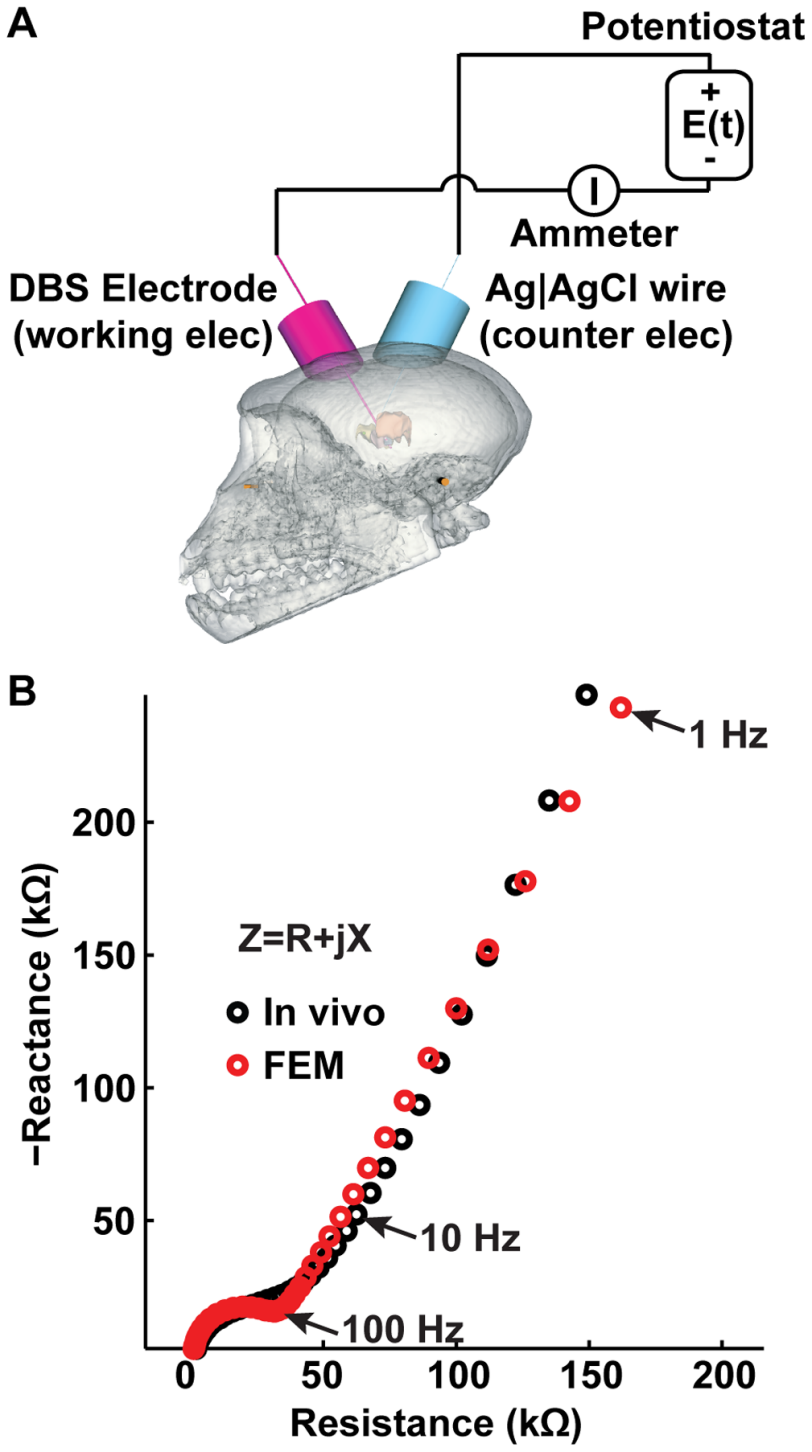
Optimization of the interface layer conductivity (σ_inter_) and relative permittivity (ε_r,inter_). (A) Schematic of the two-electrode cell configuration used to measure the electrode impedance of DBS electrodes implanted in the brain of a non-human primate (see [37]). (B) An example of the impedance spectra measured in vivo (from 1 Hz to 10 kHz) and the corresponding FEM impedance spectra with the optimized values for σ_inter_ and ε_r,inter_.

Experimental measurements have shown that DBS electrode impedance is largely dominated by the impedance of the tissue immediately adjacent to the electrode surface [37]. Therefore, to mimic the variability in electrode impedance observed clinically [20], [37], [38], the conductivity (σ_inter_) and relative permittivity (ε_r,inter_) of the interface layer were estimated for both low and high electrode impedance conditions. Low impedance parameters represent the conditions of a newly implanted electrode (i.e. acute recording conditions) or an active electrode in which stimulation was being applied (i.e. clinically-therapeutic electrode). High impedance parameters represent the condition of a chronically-implanted electrode or an inactive electrode in which stimulation was not being applied. A direct-search method was used to optimize σ_inter_ and ε_r,inter_ for each impedance condition so that the FEM impedance resembled in vivo electrode impedance spectroscopy (EIS) data measured from DBS leads chronically implanted in the brain of a non-human primate (Fig. 2) [37]. For the low impedance condition, parameter optimization produced values of 0.032 S/m and 2.93×10^4^ for σ_inter_ and ε_r,inter_, respectively. For the high impedance condition, parameter optimization produced values of 0.004 S/m and 4.36×10^4^ for σ_inter_ and ε_r,inter_, respectively (see Lempka et al. [37] for details on the EIS data and parameter optimization).

The bulk brain tissue outside the 100 µm-thick interface layer was assigned an electrical conductivity (σ_brain_) of 0.3 S/m and a relative permeability (ε_r,brain_) of 1×10^6^ [33], [39].

#### Electrical source model

The electrical source component of the model system consisted of multi-compartment cable models of STN projection neurons. These neuron models were based on a three-dimensional anatomical reconstruction of STN neuron morphology and the electrical behavior observed in vitro and in vivo [40], [41] (Fig. 1 B).

Excitatory and inhibitory synaptic inputs were represented as simple two-state kinetic models describing first-order binding kinetics of neurotransmitters to the postsynaptic receptor [42]. Parameters of these kinetic models were chosen to represent the excitatory and inhibitory synaptic inputs as AMPA and GABA_A_ receptors, respectively. Each dendritic and somatic compartment was given a single synaptic input. To mimic the general trend of excitatory afferent connections terminating on more distal dendrites and inhibitory afferent connections terminating on proximal dendrites and the soma [28], compartments farther than 100 µm from the soma were given excitatory inputs and compartments within 100 µm or less were given inhibitory inputs (Fig. 1 B). Synaptic currents were represented as an additional current branch in the neuron model (I_syn_ = g_syn_ (V_m_ – E_syn_)). The excitatory AMPA synapses were assigned a maximum conductance (g_max_) of 0.5 nS and a reversal potential (E_rev_) of 0 mV and the parameters for the inhibitory GABA_A_ synapses were g_max_ = 0.5 nS and E_rev_ = −80 mV. For a complete description of the synaptic input model and parameters, see 42,43. The excitatory synapses were intended to represent excitatory inputs from the motor cortex as part of the hyperdirect pathway while the inhibitory synapses were intended to represent inhibitory feedback connections from the external segment of the globus pallidus [44], [45].

Synaptic inputs were generated every 50 ms on average (i.e. 20 Hz) to mimic the beta-band hypersynchrony often observed in the Parkinsonian state [2]–[4]. Temporal jitter was added to the mean synaptic inputs of the individual neurons by shifting them about the 20 Hz population mean with values stochastically chosen for a truncated normal distribution with a standard deviation of 6.25 ms. The probability distribution was truncated or set to zero for values smaller or larger than two times the standard deviation from the mean and resulted in an overall firing duration of 25 ms for the population [31]. The onset times of synaptic inputs to the individual compartments for a particular neuron were also separated in time by stochastically selecting from a normal distribution with a standard deviation of √6.25 ms about the mean synaptic time for the individual neuron [31]. Simulations of the STN projection neuron were performed using NEURON v7.1 within the Python programming environment with a time step of 10 µs [46].

#### Electrode-electrolyte interface and recording electronics

**Figure 3:**
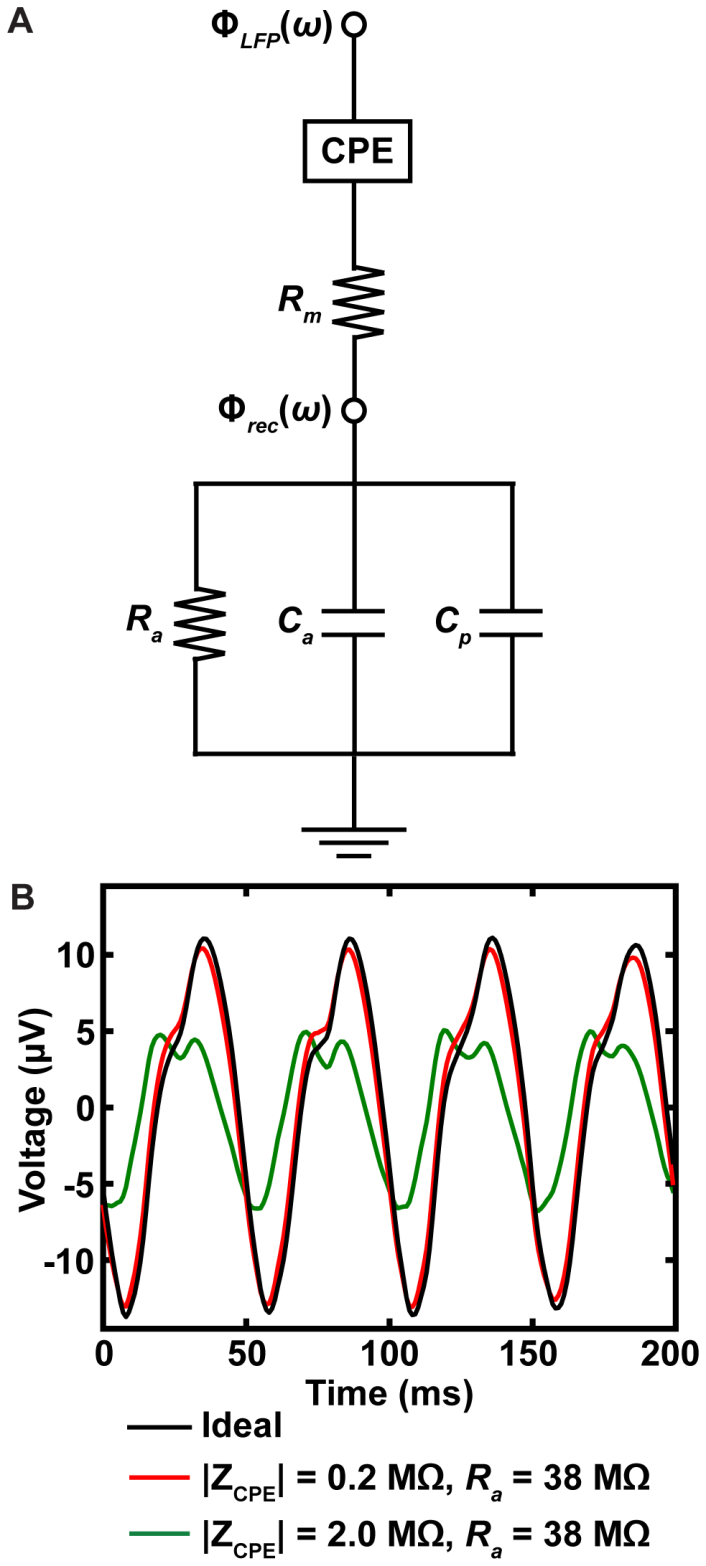
Effects of the EEI and recording electronics on LFP composition. (A) The diagram represents the circuit used to examine the effects of the EEI and the recording electronics on the composition of the LFP recording. The input to the overall circuit, Φ_LFP_(ω), was the voltage solution from the FEM (see Eq. 5). The EEI impedance was represented by a constant phase element (CPE) that accounts for the non-ideal capacitance of solid metal electrodes (see Eq. 1). R_m_ represented the resistance of the metal wires from the electrode to the recording electronics. C_p_ represented possible parasitic capacitance of the electrode shaft and wires. The resistance and capacitance of the recording head-stage were represented with R_a_ and C_a_, respectively. Φ_rec_(ω) represented the voltage recorded at the head-stage (see Eqs. 6–7). (B) The plot shows the LFP recorded with microelectrodes having impedance magnitudes of 0.2 and 2 MΩ at 1 kHz with a low-input impedance head-stage (i.e. R_a_ = 38 MΩ) relative to an ideal recording system with an infinite input impedance. The neural population consisted of a sphere with a radius of 5 mm (see methods). The LFPs recorded with a high-input impedance head-stage were not shown due to the small differences relative to the LFP recorded with an ideal recording system.

The effects of the electrode-electrolyte interface (EEI) and the recording electronics were also incorporated into the recording model (Fig. 3 A). The EEI was modeled as a constant phase element (CPE) represented by the following equation:
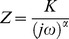
(1)in which K was a magnitude scaling factor and α was a phase factor defined for 0≤ α ≤1. The CPE represents the non-ideal capacitive behavior of the EEI due to surface roughness and specific adsorption effects [47]. For the DBS electrode, K and α were determined by fitting Eq. 1 to in vitro EIS data of a Medtronic DBS lead using nonlinear weighted least squares and function weighting (see Eq. 2 in [37]). The parameter optimization produced values of 2.02×10^5^ Ωs^-α^ and 0.87 for K and α, respectively. For the intraoperative recording microelectrode, K was set to either 0.41×10^9^ or 4.07×10^9^ Ωs^-α^ and α to 0.87 with corresponding 1 kHz impedance magnitudes of 0.2 and 2.0 MΩ representing a typical range for intraoperative recording microelectrodes [48].

We accounted for the resistance of the metal wires leading from the DBS lead and microelectrode (R_m_) with a resistance of 40 Ω [49]. Possible parasitic capacitance (C_p_) was also incorporated into the model using a capacitance of 20 pF and 2.7 pF for the DBS lead and microelectrode, respectively [37], [50]. C_p_ represents capacitance across the insulation between the electrode shaft and the surrounding electrolyte, capacitance between adjacent electrodes (for the DBS lead), as well as accumulative capacitance across the cables connecting the electrode to the recording head-stage. The input impedance of the recording head-stage was represented as a parallel resistance (R_a_) and capacitance (C_a_). Multiple head-stage input impedances were examined to investigate potential effects of the EEI and recording electronics on the composition of the LFP recordings. Low and high input impedance values of the recording head-stage were considered as *R_a_* = 38 MΩ, *C_a_* = 3 pF and *R_a_* = 1 GΩ, *C_a_* = 2 pF, respectively [50].

#### Model coupling

To simulate LFP recordings in the brain, the volume conductor model and electrical source models were coupled mathematically using a reciprocity-based solution [23], [51]. In the coupled FEM-neuron model, each neural compartment was represented as an independent current source (i.e. the time-dependent transmembrane currents computed in NEURON) at the appropriate spatial location in the FEM. The overall voltage at the recording electrode was calculated by superimposing the voltages generated at the electrode by the transmembrane currents of the individual neural compartments.

The fundamental task was to calculate the voltage impressed at the electrode for a given current at an arbitrary point in the volume conductor. For an electrostatic solution, this can be formulated mathematically with the following expression:
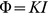
(2)where Φ is a (1×t) vector containing the voltage recorded at t instances in time, K is a (1×j) vector containing the voltages that would be impressed at the recording electrode for a unit current at the location of each of the j individual neuron compartments, and I is a (j×t) matrix containing the transmembrane currents for the individual neural compartments at each time step. The I matrix was calculated in NEURON, while each value in the K vector was derived from the FEM using a reciprocal solution. Briefly, this reciprocal solution involved placing a unit current source (i.e. 1 A) at the recording electrode and solving for the scalar potentials generated at each node in the volume conductor mesh. By the theorem of reciprocity, the voltage at a given node in the mesh can be interpreted as the voltage that would be generated at the recording electrode for a unit current. Therefore, the contribution of each neural compartment to the recorded waveform (i.e. individual values in the K vector) could be calculated using interpolation of the voltages from the nearest nodes surrounding each neuronal compartment.

While electrostatic solutions only required a single reciprocal solution (i.e. only one *K* value for each compartment), electrodynamic solutions required the FEM to be solved at each frequency (i.e. different *K* value at each of the desired frequencies). However, the fundamental principles of the reciprocity-based approach for electrostatic solutions described above remained the same for electrodynamic solutions. Electrodynamic solutions also required taking the fast Fourier transform (FFT) of the transmembrane currents, i_j_(t), for each neural compartment:
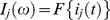
(3)


Frequency-dependent reciprocal solutions were generated by placing a sinusoidal 1 A current source with the desired frequency at the recording electrode. The LFP signal generated by an individual neuron was calculated by scaling the reciprocal solutions at each frequency, *K_j_*(*ω*), according to *I_j_*(*ω*) for each compartment and summing the voltages together (k compartments total):
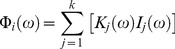
(4)In Eq. 4, Φ_i_(ω) represents the LFP signal generated by neuron, i, in the frequency domain. The overall LFP at the recording electrode generated by multiple neurons (n total neurons) was then calculated in the frequency domain by summing the LFP signals generated by the individual neurons.



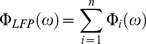
(5)The recorded LFP signal was then estimated by calculating the transfer function of the circuit shown in Fig. 3 at each of the solution frequencies. The recorded LFP was calculated according to the following:
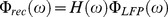
(6)where H(ω) is the transfer function describing the voltage at the recording head-stage:
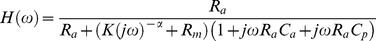
(7)Φrec(ω) was solved at each frequency determined by the LFP recording parameters described below and the corresponding time domain signal derived from the inverse FFT. The recorded LFP signal was also band-pass filtered from 1–100 Hz with a two-pole high-pass and a two-pole low-pass Butterworth filters.

In the coupled FEM-neuron model, neurons were placed 200 µm apart, representing a neuronal density of 125 neurons/mm^3^. Neurons with compartments intersecting the recording electrode were removed from the simulation. Transmembrane current solutions of 1 s in duration were generated in NEURON and then downsampled to a sampling rate of 1 kHz, providing a 1 Hz frequency resolution in the FFT of the neural data with a maximum frequency of 500 Hz. Power analysis was performed by calculating the modified periodogram with a Hamming window. LFP amplitude was calculated as the standard deviation of the recorded signal and the LFP ‘spatial reach’ was defined as the distance at which the LFP amplitude had reached 95% of its maximum value [33].

## Results

### Model Complexity

This study implemented detailed models of LFP recordings to ensure accuracy and also determine the effect of model complexity on LFP simulations. The recording model accounted for the frequency-dependent properties of the bulk brain tissue, inhomogeneities in the tissue local to the recording electrode, the electrode-electrolyte interface (EEI), and the recording electronics. To determine the necessary components, several models of monopolar recording with a DBS electrode were constructed that employed various levels of complexity. LFP recordings for each model were generated for a spherical population of neurons with a radius of 5 mm that contained a total of 62,276 neurons.

To determine the necessary complexity of the FEM, solutions were generated for the following model types: electrodynamic FEMs for both low and high interface layer impedance; electrodynamic FEMs with no bulk brain capacitance for both low and high interface layer impedance; and electrostatic FEMs for both low and high interface layer impedance (six models in total). The maximum difference in LFP amplitude between all of the model types was on the order of 3.03%. These small differences suggest the capacitance of the interface layer and bulk brain tissue, and the low and high impedance conditions of the interface layer had no significant effect on the recorded LFP. Therefore, the remaining analyses in this paper utilized electrostatic solutions of the volume conductor model with σ_inter_ = 0.032 S/m and σ_brain_ = 0.3 S/m.

To determine the importance of the EEI and recording electronics, we evaluated the following combinations for both the DBS lead and microelectrode: EEI impedance and a low-input impedance head-stage; EEI impedance and a high-input impedance head-stage; and an ideal head-stage with an infinite input impedance. For the DBS lead, there was a 0.003% and 0.001% attenuation in LFP amplitude with a corresponding 0.005% and 0.002% decrease in beta-band power for the low- and high-input impedance head-stages relative to the ideal head-stage, respectively. These results suggest the impedance of the EEI and recording electronics had virtually no effect on the composition of the LFP signal recorded with the DBS lead.

Because microelectrode impedance can be highly variable, we examined the effects of the EEI and recording electronics for microelectrodes with 1 kHz impedance magnitudes of 0.2 and 2 MΩ, which represented a range typically observed with these clinical electrodes [48]. For the low-impedance microelectrode (i.e. 0.2 MΩ), there was a 4.71% and 0.48% attenuation in LFP amplitude with a corresponding 8.90% and 0.94% decrease in beta-band power for the low- and high-input impedance head-stages, respectively (Fig. 3 B). For the high-impedance microelectrode (i.e. 2 MΩ), there was a 50.5% and 4.73% attenuation in LFP amplitude with a corresponding 76.5% and 9.01% decrease in beta-band power for the low- and high-input impedance head-stages, respectively (Fig. 3 B). These results show it is possible for high impedance microelectrodes to produce significant distortions in the recorded LFP signal. However, the results also show that these distortions can be minimized with a lower microelectrode impedance and/or recording electronics with a sufficiently high input impedance. Therefore, the effects of the EEI and recording electronics were not considered in the remaining analyses presented in this study.

### Macroelectrode v. Microelectrode

**Figure 4:**
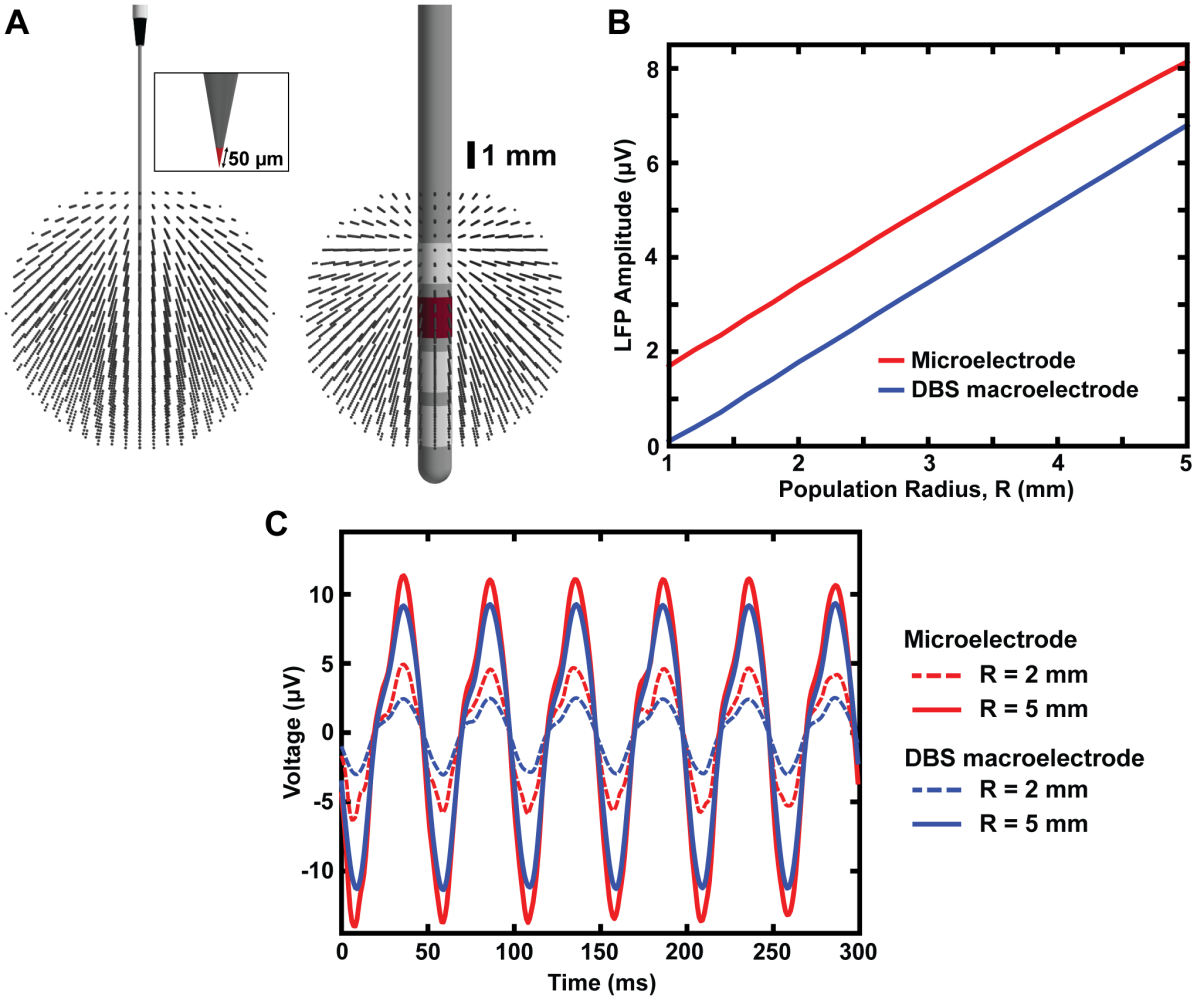
LFP recordings for microelectrodes and macroelectrodes. (A) The LFPs recorded with a microelectrode and DBS macroelectrode were compared using a spherical population with a variable radius (1≤ R ≤5 mm). Each dot represents the location of the soma of an individual STN projection neuron. For visualization purposes, a reduced cell density is shown above (i.e. 8 neurons/mm^3^). LFPs recorded with the intraoperative microelectrode were calculated as the difference between the microelectrode tip and the reference electrode. The inset shows a close-up of the microelectrode tip and the 50-µm tip exposure. The 1 mm tall reference electrode can also been seen at the distal end of the cannula. Monopolar LFPs recorded with the DBS macroelectrode were calculated with the outer boundaries of the FEM set to ground (see methods). (B) The LFP amplitude as a function of population radius for both the microelectrode and macroelectrode. (C) Model LFP recordings for both of the electrode types with R = 2 mm (dashed lines) and R = 5 mm (solid lines).

In this study, model solutions were generated for both an intraoperative microelectrode and a DBS macroelectrode, allowing us to compare the LFPs recorded with both electrode types. This comparison was performed with a spherical volume of neurons with a variable population radius, R (1 mm ≤ R ≤5 mm) (Fig. 4 A). For R = 5 mm, the populations consisted of 65,153 and 62,276 neurons for the microelectrode and DBS electrode, respectively. The microelectrode LFP recording was calculated as the difference between the solutions for the microelectrode tip and the reference electrode at the distal end of the cannula (Fig. 4 A). For the DBS electrode, a monopolar recording was estimated with the outer bounds of the FEM set to ground. Recordings with the microelectrode and DBS electrode both showed a linear increase in LFP amplitude as a function of R (Fig. 4 B). While the LFP from both electrode types was similar in shape, the microelectrode recordings had an overall higher amplitude (Fig. 4 *C*).

### Recording Configuration

**Figure 5:**
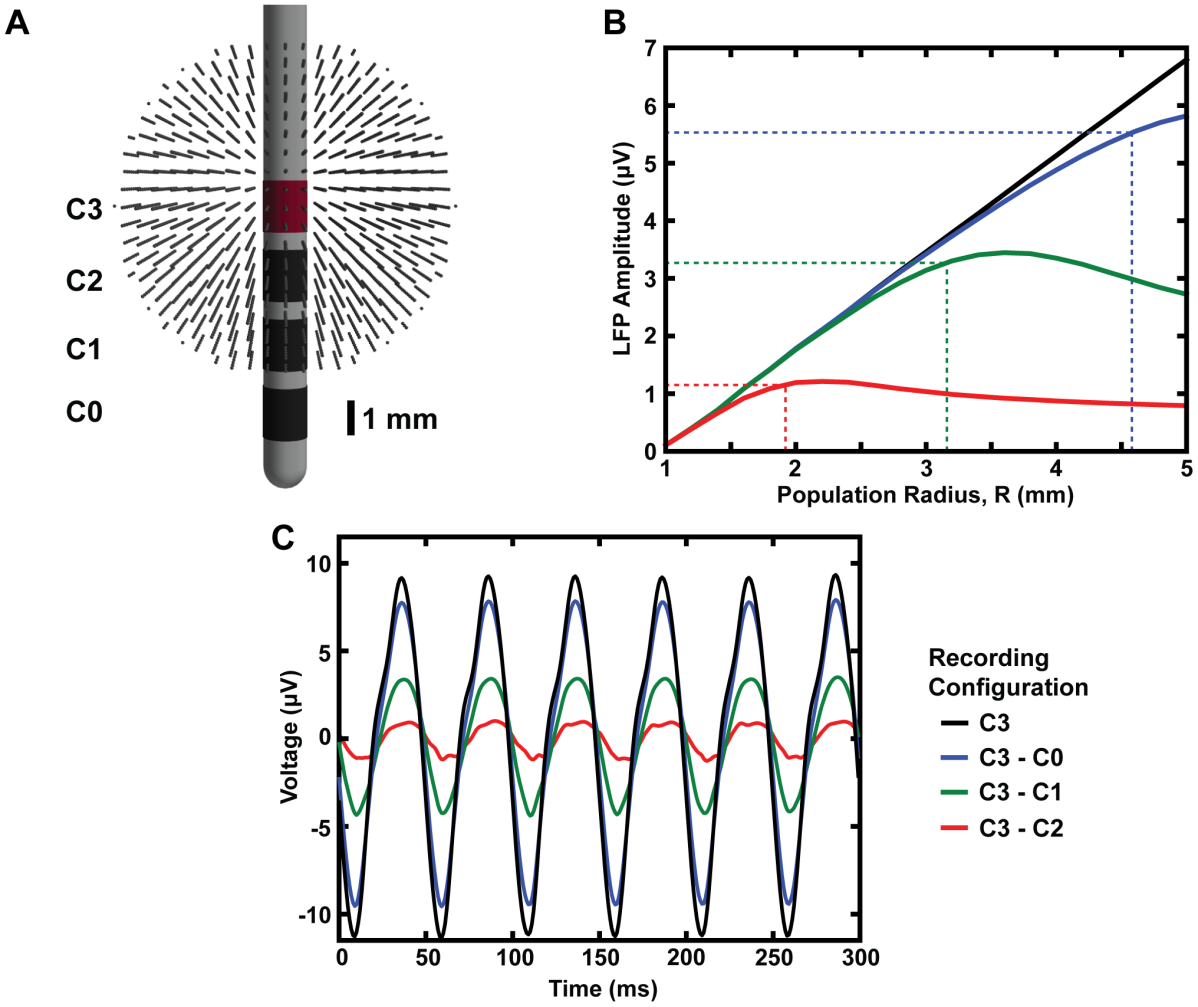
Recording configuration effects on the LFP. (A) Recording configuration effects on the LFP were examined using a spherical population with a variable radius (1≤ R ≤5 mm) centered around electrode, C3. Each dot represents the location of the soma of an individual STN projection neuron. For visualization purposes, a reduced cell density is shown above (i.e. 8 neurons/mm^3^). Four different recording configurations were considered: monopolar (C3), and three bipolar configurations (C3–C0, C3–C1, C3–C2). (B) The LFP amplitude as a function of population radius for all four recording configurations. The dashed lines represent the spatial reach of the LFP (defined as the distance at which the LFP reached 95% of its maximum) for each recording configuration. (C) LFP recordings for all four recording configurations with R = 5 mm.

The recording model was also used to examine the effects of recording configuration on the composition of the LFP signal. For LFPs recorded with the DBS lead, four different recording configurations were compared. These configurations consisted of monopolar along with three bipolar recording configurations with the negative recording electrode located at various distances from the positive recording electrode (Fig. 5 A). Bipolar simulations were calculated by taking the difference between the monopolar solutions for the positive and negative recording electrodes. For the monopolar recording configuration, the LFP amplitude increased with an increase in the population radius (R) and did not converge to a fixed value (Figs. 4 A and 5 A); however, for bipolar recording configurations, the reach of the LFP began to converge and was directly related to the distance between the positive and negative recording electrodes. For the three bipolar recording configurations: C3–C0, C3– C1, and C3– C2, the LFP had a reach of 4.6, 3.2, and 1.9 mm and corresponding interelectrode spacings of 4.5, 2.5, and 0.5 mm, respectively (Fig. 5 B). The recording configuration not only affected the reach of the LFP, but also the maximum LFP amplitude. The recording configurations: C3– C0, C3– C1, and C3– C2 had maximal LFP amplitudes on the order of 5.8, 3.4, and 1.2 µV respectively. Bipolar recording configurations also produced non-monotonic changes in the LFP amplitude. As the population radius (R) increased, the LFP amplitude initially increased, reached a maximum amplitude, and then began to decrease due to neurons located more closely to the negative recording electrode (Fig. 5 B).

### Correlations in Synaptic Activity

**Figure 6:**
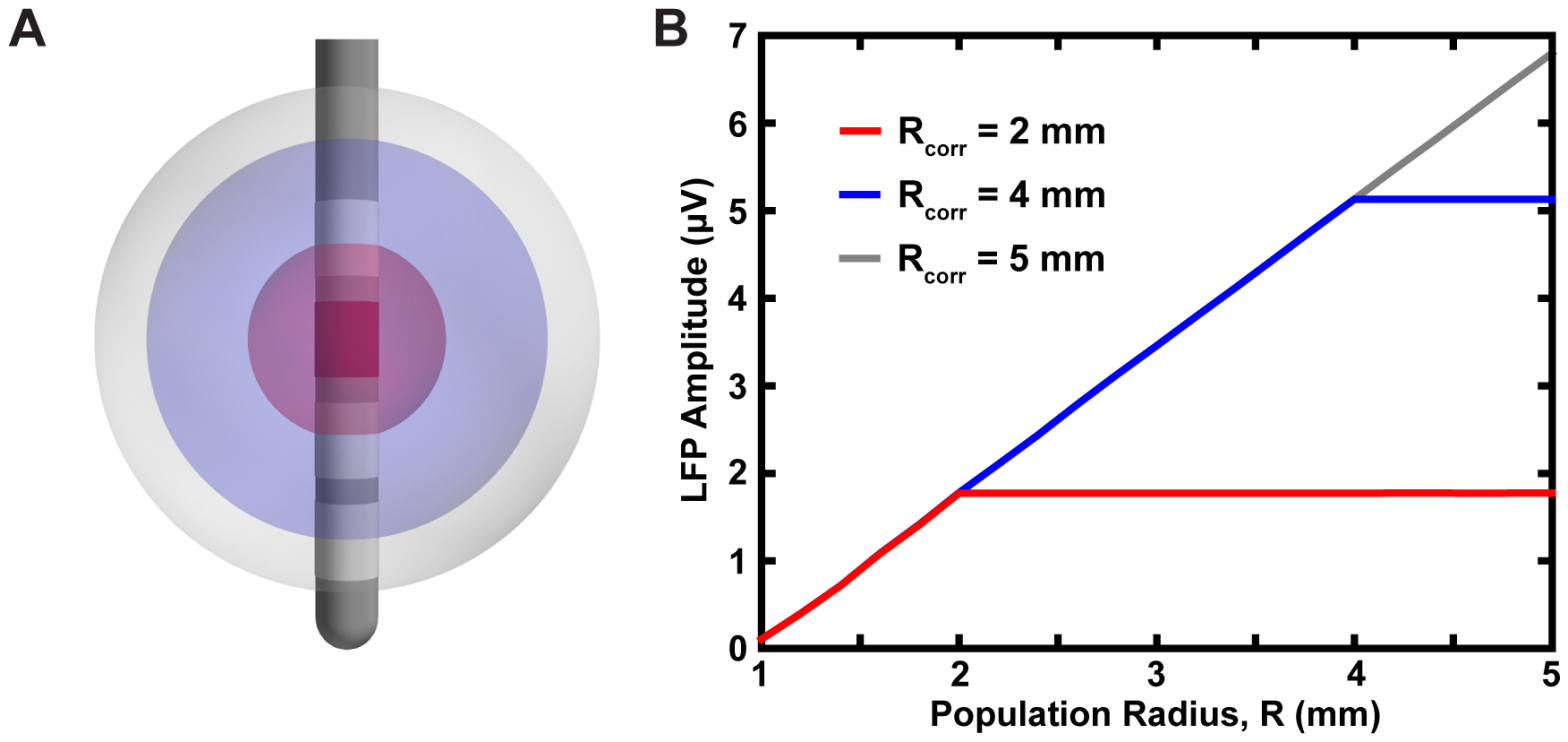
Correlated synaptic activity dominates the LFP and determines its spatial reach. (A) Monopolar LFP recordings were estimated from a spherical population of neurons with an overall radius of 5 mm. Three different volumes of correlated activity were considered, R_corr_ = 2, 4, and 5 mm. (B) The LFP amplitude as a function of population radius for all three values of R_corr_.

Theoretical analyses of the spatial reach of the LFP suggest it is determined by the degree and spatial extent of correlations in synaptic inputs [33]. In the analyses described above, the entire population of neurons was assumed to be highly correlated. To determine the effect of correlations in synaptic activity on the LFP spatial reach, correlated synaptic activity was restricted to a sphere with a specific radius, R_corr_. Neurons located at a distance greater than R_corr_, received synaptic inputs that were uncorrelated. For uncorrelated synaptic activity, the input times of individual somatic and dendritic compartments were determined by independent Poisson processes with an average rate of 20 Hz. Monopolar recordings with a DBS lead were simulated for an overall spherical population of neurons (radius of 5 mm) and three different volumes of correlated activity R_corr_ = 2, 4, and 5 mm. Within correlated regions (R ≤ R_corr_), an increase in the population radius, R, produced a linear increase in the LFP amplitude (Fig. 6 B). Outside the correlated volumes (R>R_corr_), there was no significant increase in LFP amplitude (Fig. 6 B). These results show that correlated synaptic activity dominates the LFP and its spatial reach is determined by the size of the hypersynchronous region.

## Discussion

### Filtering Effects of the Brain, Electrode, and Recording Electronics

The goal of the study was to develop a detailed model of LFP recordings in clinical DBS applications and use this model to identify key electronic and biophysical factors that affect the recorded signals. The results of this study show that a number of assumptions can be made that simplify the model infrastructure while still generating accurate recordings of the LFP. For example, to minimize distortion of the recorded LFP signal, it is necessary to select appropriate recording electronics [50]. To examine possible effects of the EEI and the recording electronics on the composition of the recorded LFP, the LFP generated by the FEM (see Eq. 5) was applied to the circuit shown in Fig. 3 A (see Eqs. 6–7). Parameters for the circuit in Fig. 3 A were selected to mimic recording head-stages with both low and high input impedances [50]. The results showed that the EEI and recording head-stage impedance produced virtually no difference in the LFP recorded with DBS macroelectrodes. However, studies have shown it is possible for the microelectrode EEI to produce distortions in the recorded LFP [50], [52]. In this study, we considered the range of 0.2 to 2 MΩ impedance magnitudes at 1 kHz, which represented a typical range for these clinical microelectrodes [48]. The results showed that at 2 MΩ it was possible for significant LFP distortions to occur with recording electronics that had a lower input impedance. Some investigators also utilize microelectrodes with higher impedances (≤10 MΩ) that can lead to significant distortions in the LFP, even for recording electronics with a high input impedance (data not shown) [48].

We also considered low-pass filtering effects due to the tissue capacitance. Much of the prior literature suggests the spatial reach of the LFP is frequency dependent due to both the low-pass filtering effects of biological tissue and neuron morphology [32], [34]. However, the work of Kajikawa et al. [30] and Logothetis et al. [53] suggest the impedance of brain tissue is largely resistive and can be assumed to be frequency-independent. To examine the potential low-pass filtering of the LFP due to the biological tissue, non-zero permittivities were assigned to both the interface layer and the bulk brain tissue. Our simulated LFP results showed there was virtually no difference between a electrodynamic solution and the corresponding electrostatic solution. Therefore, we concluded the frequency-dependent properties of the brain tissue do not affect the composition of the recorded LFP.

To mimic the variations in electrode impedance observed clinically [20], [37], [38], the impedance of the tissue immediately adjacent to the recording electrode was also varied between low and high impedance conditions based on experimental measurements [37]. The low electrode-tissue interface impedance was intended to represent the impedance observed for an acutely-implanted electrode due to edema or an active electrode in which stimulation is chronically applied. The high electrode-tissue interface impedance was intended to represent the impedance of a chronically-implanted electrode due to the foreign body reaction around an inactive electrode. The model results showed virtually no change in the recorded LFP as a function of electrode-tissue interface impedance. This result mimicked the trend observed experimentally in which changes in DBS electrode impedance were not correlated with changes in the beta-band power of the LFP [38].

### Spatial Scale of the LFP

Numerous clinical and experimental investigations are currently underway to evaluate if LFP recordings can be used to localize nuclei of the BG, assist in surgical target identification, optimize DBS electrode placement, and serve as a control signal for closed-loop control of DBS systems [14]–[18], [22], [54]. Recent studies have shown the spatial extent of hypersynchronous neural activity was correlated with the severity of certain PD motor symptoms (e.g. bradykinesia and rigidity) and the therapeutic efficacy of DBS [13], [14]. These results suggest selection of an electrode trajectory for implanting the DBS lead that maximizes the span of the synchronous region [14].

Successful implementation of LFP recordings in these applications largely relies on an adequate knowledge of the LFP spatial reach. In this study, we compared the LFP recorded with both microelectrodes and macroelectrodes and how the LFP changes as function of population size (population radius = R). While the intraoperative microelectrode had higher recording amplitude relative to the DBS macroelectrode, an increase in R produced a linear increase in LFP amplitude for both electrode types (Fig. 4 B). This result is in agreement with recent experimental and theoretical studies and suggests it is possible for LFP recordings to be sensitive to the volume conduction of neural signals several millimeters from the recording electrode [29], [30], [33]. The linear increase in LFP amplitude as a function of population size can be easily explained. If an individual neuron is approximated as an electric dipole source, the amplitude of the voltage it would contribute to the LFP recording would decrease at a rate proportional to 1/r^2^ (where r is the distance between the neuron and the recording electrode). However, this decrease in amplitude is compensated for by the increase in the number of neurons as R increases. In this study, we used spherical populations with a constant neural density (i.e. 125 neuron/mm^3^) in which the number of neurons would increase at a rate directly proportional to R^2^. Therefore, the approximate overall rate of change in LFP amplitude as a function of R is constant.

This linear increase in LFP amplitude as a function of R was observed for a monopolar recording configuration using a distant reference electrode (i.e. the outer boundaries of the FEM were set to ground). Because it is possible for the LFP to extend several millimeters, a main concern in LFP analysis is that a distant reference electrode can lead to uncertainty in the neural tissue responsible for generation of the LFP. Therefore, differential recording methods (e.g. current source density analysis and bipolar recordings) are often utilized to limit the spatial scale of the LFP by eliminating far-field volume conduction effects [13], [28], [30], [31]. To investigate the effect of recording configuration on the amplitude and spatial reach of the LFP, we examined multiple recording configurations with the DBS macroelectrode (Fig. 5). Bipolar recordings produced a significant decrease in the LFP amplitude and resulted in a finite LFP spatial reach. Therefore, bipolar configurations should decrease the sensitivity to distant neural sources and allow the recording to be dominated by neurons local to the recording electrodes. Bipolar recording configurations should also improve the specificity of the DBS macroelectrodes in localizing nuclei, mapping regions of hypersynchrony within a particular nucleus, and studying therapeutic changes in neural activity near the electrodes.

Previous work has shown that the spatial scale of the LFP is largely determined by the correlations in synaptic activity [33]. Therefore, an additional goal of this study was to examine the importance of correlated synaptic activity in determining the spatial reach of the LFP recorded in DBS applications. Similar to [33], our results show that the LFP is dominated by neurons with correlated synaptic inputs and its reach is determined by the size of the correlated region (Fig. 6 B). This result has significant clinical and experimental implications because it suggests the spatial reach of the LFP will, in general, not be static within a given experiment. For example, drug treatments or electrical stimulation that produce changes in neural activity near the recording electrode will not just alter the LFP power spectrum, but they will also affect the spatial scale of the LFP.

### Study Limitations and Future Work

Although the model presented in this study provides a substantial advancement in technical detail for LFP recording models, the analysis was subject to a number of limitations. First, our neuron density of 125 neurons/mm^3^ was much lower than the total neuron density in the STN (e.g. 1370 neurons/mm^3^) [55]. This decreased neural density was chosen to help decrease the computational demands of the model analysis and is not likely to affect the observed trends. Due to the linear nature of the FEM utilized in this study, changes in the neuron density would produce linear changes in the absolute amplitude of the LFP (e.g. a two-fold increase in the neuron density would produce a two-fold increase in the absolute LFP amplitude) (data not shown). Another limitation was that the neural source utilized in this study only accounted for postsynaptic currents of the simulated synaptic inputs. Both theoretical and experimental measurements suggest the recorded LFP can contain contributions from both presynaptic (i.e. afferent terminal discharge) and postsynaptic mechanisms; however, the presynaptic component is typically masked by the much larger postsynaptic component [30]. In addition, the compartmental model included active membrane properties which represented an improvement upon several previous theoretical studies [31]–[33]. Active membrane properties can be important in modeling the LFP because the synchronous action potentials of several neurons can contribute to the high-frequency content of the LFP [56], [57].

The results of this study show that a frequency-dependent volume conductor does not produce significant differences in the simulated LFP. However, the volume conductor model implemented in this study was largely homogeneous except for the inhomogeneity of the 100-µm thick interface layer near the electrode. Our largely homogeneous volume conductor could decrease the LFP frequency dependence because inhomogeneities in the extracellular media are necessary to produce frequency-dependent properties [34]. We also ignored the possible contributions of ionic diffusion in producing frequency-dependent volume conduction of the LFP. Transmembrane currents represent a flux of ions across the cell membrane and local ion concentrations must be maintained by a net diffusion of ions. It has been suggested that this ionic diffusion is responsible for the 1/f structure of the LFP signal that is observed experimentally [34]. In this study, we focused our analysis on recording hypersynchronous activity within the beta frequency range (13–30 Hz) because this frequency range has been studied extensively and experimental results suggests it is related to the motor state of patients with Parkinson’s disease [2], [22], [54]. However, oscillatory activity in other frequency bands, such as gamma (35–100 Hz) or high-frequency (∼300 Hz), have also been observed within the STN [58], [59]. At these higher frequencies, it is possible that the capacitive properties of brain tissue could promote frequency-dependent attenuation in the LFP [34]. However, experimental evidence suggests volume conduction of the LFP is largely frequency-independent and any frequency-specific effects would more likely be attributed to the larger effects temporal variations have on the coherence of high-frequency relative to low-frequency signals [30].

The purpose of this work was to examine a number of biological and technical factors that can affect the composition of the LFP. These variables included: electrode geometry, electrode impedance, recording configuration, and potential filtering effects of the brain, electrode-tissue interface, and the recording electronics. Although we believe the results of this study can be applied to other clinical and basic science applications, it is important to consider model parameters that may lead to potential limitations in the general applicability of the results. The origin of the LFP is complex in nature and changes in a large number of parameters can lead to differences in the shape and/or amplitude of the LFP. While several of these parameters (e.g. rate of synaptic input, cell density, ion channel kinetics, anisotropic tissue conductivity) can produce differences in the amplitude and/or shape of the LFP, they will likely not affect the spatial scale of the LFP (data not shown). As this study and previous studies have shown, factors that determine the spatial scale of the LFP include: recording configuration, neuron geometry (i.e. closed-field v. open-field), neuron orientation relative to the recording electrode, distribution of synaptic inputs, and correlations in synaptic activity (Figs. 5 and 6) [33]. Detailed characterization of some of these variables was beyond the scope of this study; however, such investigations will represent interesting and important next steps in the theoretical characterization of clinical LFP recordings. This study also had an “electro-centric” view in which we used the population size to determine the spatial reach. Future studies will also include characterizations from a “population-centric” view, in which the spatial reach of the LFP will be defined by how far the LFP can be detected outside of the hypersynchronous region [33].

### Conclusion

This study utilized a theoretical recording model of beta oscillations in the STN to improve our understanding of clinical LFP recordings. This recording model consisted of FEMs of recording electrodes implanted in the brain, multi-compartment models of STN projection neurons, as well as circuit elements accounting for the impedance of the EEI and the recording electronics. Model analysis showed the spatial reach of the LFP can extend several millimeters. Electrode geometry (e.g. microelectrode v. macroelectrode) and recording configuration (e.g. monopolar v. biopolar) also produced substantial differences in the LFP amplitude and spatial reach. This study illustrates that a large number of variables are responsible for the complex origin of the recorded LFP and these variables should be considered when using LFP results in clinical DBS applications.
